# Motion-compensated spin-echo cardiac diffusion tensor imaging in multiple cardiac phases using an ultrahigh gradient strength scanner

**DOI:** 10.1016/j.jocmr.2026.102699

**Published:** 2026-01-29

**Authors:** Shubhajit Paul, Camila Munoz, Pedro F. Ferreira, C.John Evans, Sonya Foley, Fabrizio Fasano, Derek K. Jones, Dudley J. Pennell, Sonia Nielles-Vallespin, Andrew D. Scott

**Affiliations:** a Cardiovascular Magnetic Resonance Unit, Royal Brompton Hospital, Guy’s and St Thomas’ NHS Foundation Trust, London, UK; b National Heart and Lung Institute, Imperial College London, London, UK; c Cardiff University Brain Research Imaging Centre (CUBRIC), Cardiff University, Cardiff, UK; d Siemens Healthcare Ltd, Camberly, UK; e Siemens Healthcare GmbH, Erlangen, Germany

**Keywords:** Cardiac diffusion tensor, High gradient strength, Motion-compensated diffusion encoding

## Abstract

**Background:**

Cardiac diffusion tensor imaging (cDTI) has traditionally relied on inefficient stimulated echo techniques to robustly assess microstructural changes over the cardiac cycle. Ultrahigh gradient strength systems (>80 mT/m) allow shorter motion-compensated diffusion encoding. This study compares the ability of high and ultrahigh strength gradient systems to provide systolic and diastolic motion-compensated spin echo (MCSE) cDTI.

**Methods:**

Second-order MCSE sequences were developed for a research-only Siemens 3T Connectom (300 mT/m maximum gradient amplitude per axis), and breath-hold cDTI was acquired at peak systole and end diastole. Acquisitions used the maximum achievable gradient strength (G_UH_, 116 mT/m) and also limited to typical high gradient strengths (G_H_, 66 mT/m based on 80mT/m maximum allowable), giving TE = 48 ms and 58 ms, respectively. Data were acquired at 2.8 × 2 .8 × 8 mm^3^, b = 500 s/mm^2^ (8 averages) and b = 150 s/mm^2^ (2 averages) in 6 encoding directions.

**Results:**

Twenty-two healthy subjects were recruited. 20/21 and 21/22 systolic acquisitions at G_UH_ and G_H_, respectively, met the >50% criteria of the circumferential myocardium showing the expected transmural variation in helix angle. For G_UH_ and G_H_ (16/20) 80% and (16/22) 73% of diastolic acquisitions were successful, respectively. SNR was increased using G_UH_ compared to G_H_ (median [IQR]: 12.9 [3.8] vs. 9.6 [2.9], p = 0.0002 diastole, 15.6 [5.9] vs. 12.5 [6.7], p = 0.006 systole). Using G_UH_ fractional anisotropy was lower in systole (0.349 [0.040] vs. 0.373 [0.019], p = 0.002) and G_UH_ transmural helix angle gradient (HAG) was steeper in diastole (−0.70 [0.17] vs. −0.55 [0.12] ˚/%, p = 0.04). At both G_UH_ and G_H_, sheetlet angle (|E2A|) was higher in systole than in diastole (30.7 [7.3] vs. 21.3 [6.7]˚ p = 10^−4^ and 32.6 [10.9] vs. 26.0 [7.4]˚, p = 0.03, respectively). Differences in HAG between phases were only apparent with G_H_ (−0.88 [0.23] vs. −0.55 [0.15], p = 10^−4^) and differences in the mean diffusivity only with G_UH_ (1.64 [0.11] vs. 1.52 [0.24] ×10^−3^mm^2^/s, p = 0.002).

**Conclusion:**

Ultrahigh-strength gradient systems deliver higher SNR for MCSE and more robust imaging in diastole. While further work is required to further improve the reliability in diastole, at ultrahigh gradient strengths, cDTI using MCSE can identify dynamic changes in the cardiac microstructure. These findings will lead to more widespread use of multiphase MCSE in cDTI clinical research.

## Graphical abstract

**Figure:**
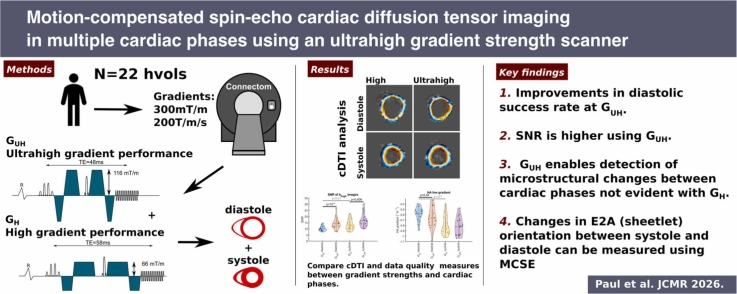

## 1 Background

Cardiac diffusion tensor imaging (cDTI) provides unique non-invasive insights into the cardiac microstructure [1], [2], [3], [4], [5]. However, detecting the displacements of water molecules diffusing tens of micrometers while the heart moves tens of millimeters with the cardiac cycle is challenging. As discussed recently, standard diffusion weighted sequences available on clinical scanners and used widely in anatomical targets outside the heart, do not currently include the required motion compensation to avoid bulk motion-related signal loss in the myocardium [6], [7]. Mitigating bulk cardiac motion in cDTI is typically achieved by either using stimulated echo techniques, which separate relatively modest diffusion encoding gradients by exactly one cardiac cycle [8], [9], [10], or by designing diffusion encoding gradients that incorporate gradient moment nulling to minimize their sensitivity to bulk motion [11], [12]. While stimulated echo acquisition mode sequences have been applied in a range of cardiovascular pathologies [2], [13], [14], [15], [16], [17], motion-compensated spin echo (MCSE) cDTI sequences have become popular due to the inefficient nature of the stimulated echo sequence and the potential for true free-breathing acquisitions using motion-compensated spin echo sequences [18], [19], [20], [21], [22], [23]. However, as we have shown previously [24], [25], [26], MCSE acquisitions frequently fail in diastolic cardiac phases. This seems counter-intuitive as the magnitude of cardiac motion is typically at a minimum during diastasis, but it seems that, over the duration of diffusion encoding, the motion trajectory of the myocardium conforms more closely to the requirements for constant acceleration (with second moment nulled waveforms) during systolic contraction than during diastasis.

Dynamic assessment of the cardiac microstructure throughout the cardiac cycle has the potential to deliver new insights into the origins of macroscopic cardiac function [3], [13], [14], [27] and inform computational models of cardiac function. Stimulated echo acquisition mode (STEAM) data acquired at peak systole and end diastole has demonstrated the ability to quantify the reorientation of the aggregates of cardiomyocytes known as sheetlets during cardiac contraction [2], [27] and the dysfunction of this process in various diseases [2], [3], [13], [14]. The STEAM cDTI signal, however, is averaged through the cardiac cycle [8], [24] and this results in a contribution to the measured signal from the strain of the tissue on the local scale of the diffusing water molecules [28], [29]. MCSE cDTI data, unaffected by strain or temporal averaging, would add value to these models of dynamic microstructure on a much smaller length scale, however, the inability of MCSE cDTI to robustly provide such dynamic measures due to poor diastolic data quality is a potential barrier to translation of cDTI to the clinic.

One approach to reducing the sensitivity of cDTI sequences to motion is to use shorter diffusion encoding gradients. High-performance gradient systems are commonly selected for MCSE cDTI studies to reduce echo times and minimize signal loss in the short T2 myocardium, with typical maximum gradient strengths of ∼80 mT/m per axis [18], [30], [31]. However, ultrahigh maximum gradient strength systems, such as the Connectom (Siemens Healthcare, Erlangen, Germany) with a maximum gradient strength of 300 mT/m per axis, may provide the opportunity to shorten motion-compensated diffusion encoding waveforms and provide more motion-robust MCSE. In this study, we hypothesize that ultrahigh-strength gradient systems (up to 300 mT/m) will facilitate more reliable and higher quality multiphase MCSE cDTI than when using high-strength gradients (up to 80 mT/m per axis) in addition to the increase in SNR available due to the shorter echo times available. In a secondary analysis, we assess whether MCSE can detect changes in microstructure as the heart contracts.

## 2 Methods

### 2.1 cDTI sequence

**Fig. 1:**
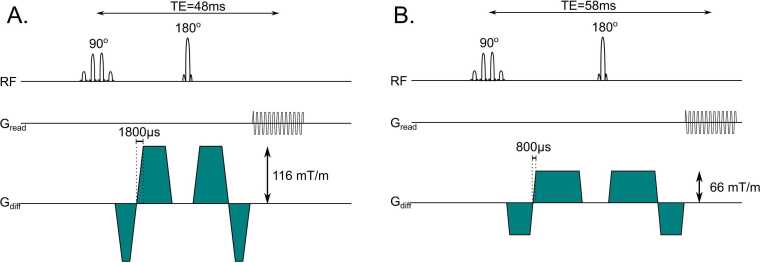
Sequences used for the G_UH_ (A) and G_H_ (B) cDTI acquisitions. The TE and gradient ramp-up/down time are labeled on the figure. Gradients are approximately to scale. See supplementary table 2 for gradient durations. Note that the 90˚ pulse is slice selective in the phase encode direction in order to reduce the size of the field of view contributing to the spin echo. *G_UH_* Ultrahigh strength gradients, *G_H_* High strength gradients, *cDTI c*ardiac diffusion tensor imaging, *TE e*cho time

An ECG-triggered second-order MCSE cDTI sequence with a single-shot EPI readout was developed for the research-only Connectom 3T scanner (Siemens Healthcare, Erlangen, Germany) based on the equal amplitude waveform design proposed by Stoeck et al. [11] and Welsh et al. [12] (Fig. 1). A binomial water-selective excitation was incorporated with initial tests demonstrating that a four sub-pulse design (1−3-3−1) provided a good balance of fat suppression with minimal TE increase. The slice select gradient applied during the sub-pulses of the 90˚ binomial pulse was used to reduce the field of view in the phase encode direction to allow shorter EPI readouts. For non-diffusion weighted images, the crusher gradients were moved as close to the 180˚ pulse as possible to reduce the duration over which the sequence is sensitive to cardiac motion, while for diffusion weighted images, the crushers were removed as the diffusion encoding gradient provided sufficient dephasing. An 18-channel body receive coil was used in combination with a 32-channel spine receive coil.

To assess the benefits of the ultrahigh strength gradient system, two encoding gradient designs were used for imaging, first using the maximum gradient strength achievable (that we refer to as ultrahigh or G_UH_). We also used a design with gradient amplitude limited to a maximum of 80 mT/m per axis (that we refer to as high or G_H_), a specification that some commercially available scanners have been able to achieve for many years. While the Connectom scanner can achieve a maximum gradient strength of 300 mT/m and slew rate of 200 T/m/s per axis, peripheral and cardiac stimulation limits the minimum gradient rise time (and therefore slew rate) [32] for a given maximum gradient strength (see Afzali et al. [33] Fig. S3 for an example). Based on our maximum b = 500 smm^−2^, we observed zero flat top for the shorter of the two diffusion encoding gradients in each pair when specifying a G_max_=180 mT/m per axis, which requires a ramp up time of 1800 µs based on the peripheral nerve stimulation limits along the most restrictive scanner axis described in Molendowska et al. [32]. Using these design constraints, we achieved G_max_ of 116 mT/m per axis when applied to two axes simultaneously (as required for our 6 direction encoding scheme) as stimulation limits prevented any further increases in G_max_. The resultant slew rate for G_UH_ was 64 T/m/s per axis. The above limits were also below the threshold for the occurrence of magnetophosphenes. This is an undesirable physiological effect possible with ultra-high gradient systems, critical on Connectom especially when the patient is positioned with the eyes far from iso-center, as is the case for cardiac imaging [32], [33]. With our G_H_ protocol, where we limited G_max_ to 80 mT/m per axis, the required ramp up time was 800 µs to avoid peripheral nerve stimulation along the most restrictive axis, and we achieved a G_max_ of 66 mT/m per axis along two axes simultaneously with a slew rate of 83 T/m/s per axis. Gradient parameters are summarized in supplementary table 1 and diffusion encoding timings are described in supplementary table 2.

### 2.2 Protocol

A short-axis slice in the mid-left ventricle was planned using bSSFP cine acquisitions. Breath-hold cDTI using the MCSE sequence was acquired in the single mid-ventricular slice four times in each subject in the following constant order: systole with G_UH_; diastole with G_UH_; systole with G_H_; diastole with G_H_. Acquisitions were ECG-triggered. Systolic acquisitions used trigger delays from the R-wave to place the central k-space lines at end-systole and diastolic acquisitions were triggered to place the diffusion encoding during the most stationary period of diastasis as defined from the short-axis cine.

Identical EPI readouts were used for all acquisitions. The readout field of view was 360 × 135 mm^2^ with a slice thickness of 8 mm and an acquired matrix size of 128 × 48 pixels, giving an acquired resolution of 2.8 × 2.8 mm^2^ in plane that was reconstructed to 1.4 × 1.4 mm^2^ on the scanner using Fourier space zero-padding. Acquisitions were triggered to alternate cardiac cycles, giving repetition time (TR) = 2 R-R intervals. Factor 2 GRAPPA parallel imaging was used to reduce the readout to 24 lines with a bandwidth 2605 Hz/px. For both G_max_ values, diffusion encoding was performed with 6 encoding directions in the fixed magnet coordinate system ([x,y,z]=[0,1,±1], [1,0,±1], [1,±1,0]) at b-values of 150 s/mm^2^ and 500 s/mm^2^. Each breath hold consisted of 9 spin echoes over 18 cardiac cycles. The first two spin echoes (4 cardiac cycles) were used to collect the EPI phase correction lines and parallel imaging reference data. The next spin echo provided the b = 0 reference image and the next 6 spin-echoes were diffusion encoded in each of the 6 encoding directions, resulting in a total breath hold duration of 18 cardiac cycles. Eight repetitions were acquired with b = 500 s/mm^2^ and two with b = 150 s/mm^2^. Total scan duration was ∼1 h per subject.

At G_UH_ TE = 48 ms and with G_H_ TE = 58 ms, which compares with TE = 62 ms using a similar protocol with G_max_= 60 mT/m per axis [26] and TE = 76 ms with G_max_= 45 mT/m per axis [24].

### 2.3 Study cohort

Twenty-two healthy volunteers were recruited with written informed consent under ethical approval from the Cardiff University School of Psychology Research Ethics Committee.

### 2.4 Analysis

cDTI data were processed using MATLAB-based tools (MathWorks, Natick, Massachusetts) developed in-house at the Royal Brompton Hospital. Diffusion-weighted images were visually inspected and those with substantial motion-induced signal loss were excluded. Images from each subject, cardiac phase, and G_max_ were registered non-rigidly using a b-spline based method in Elastix [34], [35]. Pixelwise diffusion tensors were calculated using non-linear fitting of the b = 150 s/mm^2^ and b = 500 s/mm^2^ magnitude data only. Each repetition was included separately in the tensor fitting without averaging. b = 0 data was excluded from the tensor calculation to reduce the partial volume effects in the endocardium due to the bright blood signal without diffusion weighting. The epicardial and endocardial borders were defined manually, using an estimate of the helix angle to exclude papillary muscles and trabeculations and the MD to avoid regions of very high MD caused by blood partial volume not suppressed by the non-zero b-value in the reference data. Maps of mean diffusivity (MD), fractional anisotropy (FA), helix angle (HA), absolute sheetlet angle (|E2A|), and eigenvalues were calculated. Median |E2A| and mean FA, MD, and eigenvalues were calculated across the LV myocardium, excluding papillary muscles and the right ventricular border of the LV septum. HA was quantified via the HA line gradient [36] in units of ˚ per % wall thickness.

HA map quality was assessed using a 4-point scale (0–3) [24], [25] by a blinded CMR expert with 12 years cDTI experience based on proportion of the myocardium circumferentially that displays the expected smooth progression of the HA from epi to endocardium: 0 for <50%; 1 for 50–75%; 2 for 75–95%; 3 for >95%. Datasets scoring 0 were entirely excluded from further analysis, while for datasets scoring ≥1, the whole myocardium was included in the analysis.

SNR was measured using the multiple repetitions method in the magnitude b = 500 s/mm^2^ data [10], [37] and averaged over the 6 directions. The proportion of LV myocardial pixels containing negative eigenvalues, the mean Pearson R^2^ of the linear fit to transmural HA profiles (excluding inverted gradients and R^2^ values <0.4), and the standard deviation of the transverse angle were also used as quantitative indicators of cDTI data quality.

Statistical analysis was performed in MATLAB. Parameters were calculated as the mean over the LV myocardium apart from E2A where the median absolute value was used. As parameters were found to be a mixture of normally and non-normally distributed (as assessed using a Shapiro-Wilks test), all values are presented as median [interquartile range] over the cohort for consistency. Comparisons between data acquired at the two G_max_ (G_UH_ and G_H_) at the two cardiac phases (diastole and systole) and between cardiac phases for each G_max_ are compared using a Wilcoxon sign rank test. All statistical tests used a threshold for significance of p<0.05.

## 3 Results

**Fig. 2:**
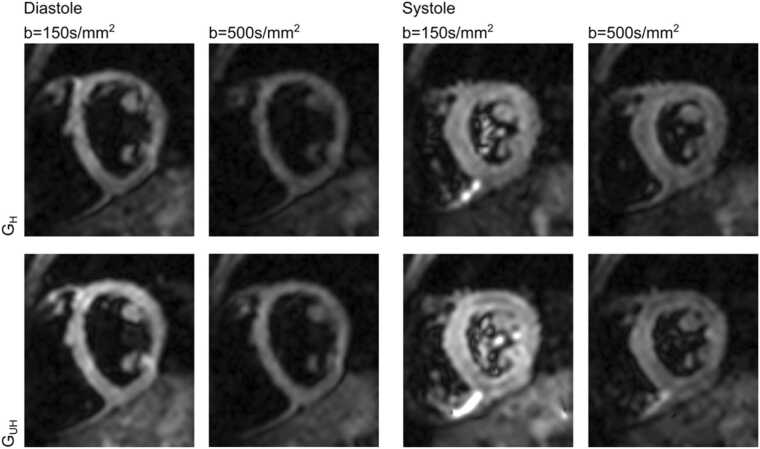
Example diffusion weighted images for one typical subject, for a single encoding direction for both gradient strengths in both cardiac phases and at both b values used in the tensor reconstruction. Images are scaled identically. SNR maps for b = 500 s/mm^2^ data from the same subject are shown in supplementary figure 1. *SNR* signal to noise ratio

Twenty-two subjects were recruited and demographics are provided in Table 1. Typical acquisition duration for each cardiac phase and G_max_ value was around 10 min. Diastolic data from 2 subjects at G_UH_ were excluded as the b = 150 s/mm^2^ was omitted from the protocol due to user error. Of the remaining diastolic acquisitions, data were excluded from further analysis or acquisition was abandoned due to severe, visible motion-related signal loss or poor quality HA maps (scoring 0) in the diffusion weighted images of 4/20 subjects (20%) for G_UH_ and 6/22 subjects (27%) for G_H_. Systolic data from one subject at G_UH_ was excluded from further analysis due to highly inconsistent breath holding and ECG mistriggering during the acquisition. Of the remaining systolic data, 1 acquisition at G_UH_ (4.8%) and 1 acquisition at G_H_ (4.6%) were excluded as the corresponding HA maps scored 0. As shown in Fig. 2, the image quality of diffusion weighted images was generally good with a clear depiction of the myocardium circumferentially at both G_UH_ and G_H_.

**Table 1:** Subject demographics

	Mean (SD) or absolute proportion [%]
Female	15 [68]
Male	7 [32]
Age (y)	24 (7)
BMI (kg m^−2^)	23.4 (3.6)
RR interval (ms)	1010 (130)

*SD* standard deviation, *BMI* body mass index.

In the cDTI analysis the median[IQR] percentage of frames visually excluded due to cardiac motion artefacts (typically motion related signal loss, see supplementary figure 3 for examples) was slightly lower for the G_UH_ data than G_H_ in diastole (0[1]% vs. 3[7]%, p=NS), but similar in systole (0[0] vs. 0[0]).

**Fig. 3:**
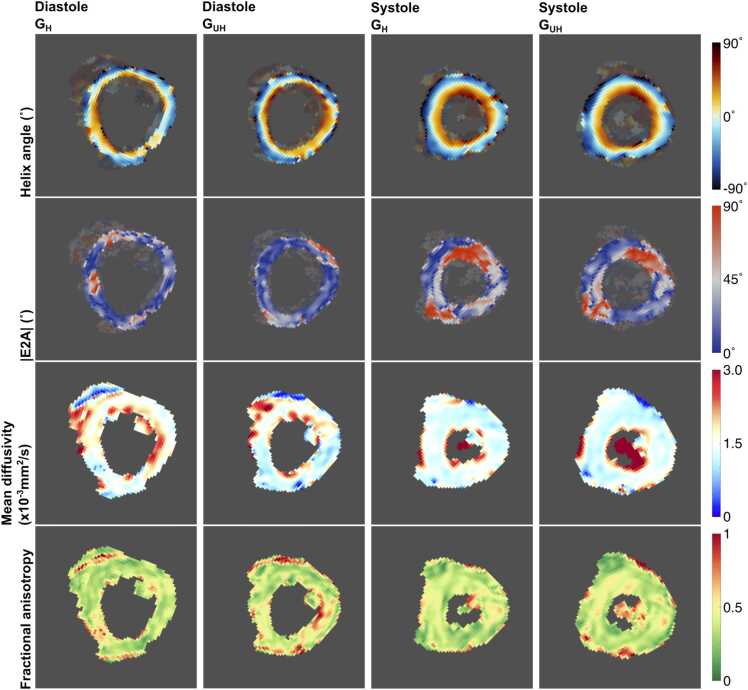
Example HA, |E2A|, MD, and FA maps for both cardiac phases and G_max_ values in one subject where all acquisitions were considered successful. Note that the LV myocardium is shown masked on the HA and |E2A| maps to demonstrate the region used in quantification. The same region was used for quantification of MD and FA, but the maps are shown unmasked. *HA* Helix angle, *E2A* absolute angle of second eigen vector, *MD* Mean diffusivity, *G_max_* Maximum gradient strength for diffusion encoding, *LV* left ventricle, *FA* Fractional anisotropy

cDTI analysis demonstrated the expected transmural variation in HA, visible increases in |E2A| at systole over diastole and suggestions of an increase in FA in the mesocardium at both G_UH_ and G_H_ (see Fig. 3).

**Fig. 4:**
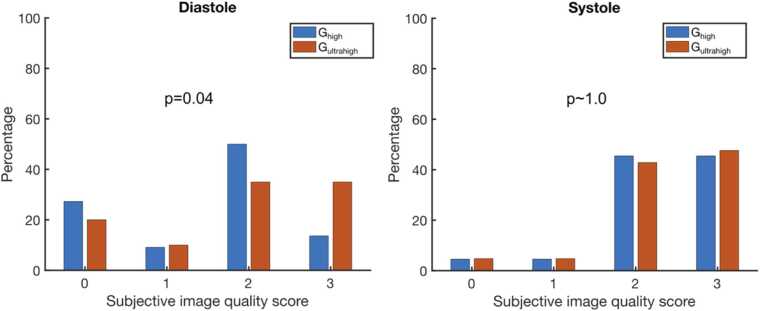
Histograms of the subjective HA scores in diastole and systole for both G_max_ values with the results of a Wilcoxon sign rank test comparing the results shown as p values. See supplementary figure 3 for examples of HA maps scored 0–3. *HA* Helix angle, *G_max_* Maximum gradient strength for diffusion encoding

A greater proportion of datasets were awarded the highest score of 3 in the subjective HA assessment (see supplementary fig. 3 for typical examples of the scores) using G_UH_ over G_H_ in diastole (7 vs. 3 in diastole), and there were fewer datasets abandoned or scored 0 in diastole using G_UH_ compared to G_H_. There was a significant increase in the image quality score between G_UH_ and G_H_ in diastole (median [IQR] scores: 2 [2] vs. 2 [2], p = 0.04) but not in systole (see Fig. 4).

**Fig. 5:**
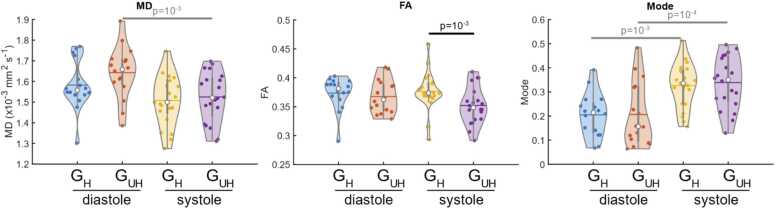
Violin plots comparing rotationally invariant cDTI parameters between the acquisitions performed with high strength (G_H_) and ultrahigh strength gradients (G_UH_) at peak systole and end diastole. The violin plots show the median as a white circle and the mean as a horizontal line. Significant differences between G_UH_ and G_H_ values are shown in black and significant differences between cardiac phases are shown in gray. *G_UH_* Ultrahigh strength gradients, *G_H_* High strength gradients, *cDTI c*ardiac diffusion tensor imaging

**Fig. 6:**
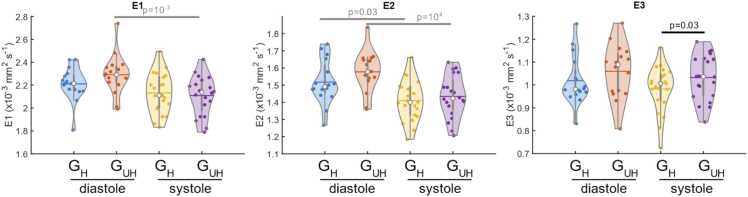
Violin plots comparing the eigenvalues for G_UH_ and G_H_ at peak systole and end diastole. See Fig. 5 for an explanation of the violin plots and p-values. *G_UH_* Ultrahigh strength gradients, *G_H_* High strength gradients

As shown in Fig. 5, MD was not significantly different between G_UH_ and G_H_ for data acquired in diastole or systole, or for FA data in diastole (for clarity, values and p-values are provided for significant results, but all values are provided in supplementary table 3). In systole, FA was significantly reduced at G_UH_ compared to G_H_ (0.349 [0.040] vs 0.373 [0.019], p = 0.002). Tensor mode [38] was not significantly different between gradient strengths. There was a significant increase in the third eigenvalue at G_UH_ compared to G_H_ in systole (1.04 [0.16] vs. 1.006 [0.098] x10^−3^ mm^2^/s, p = 0.03), but no significant differences the first or second eigenvalue between G_UH_ and G_H_ data (Fig. 6 and supplementary table 3).

**Fig. 7:**
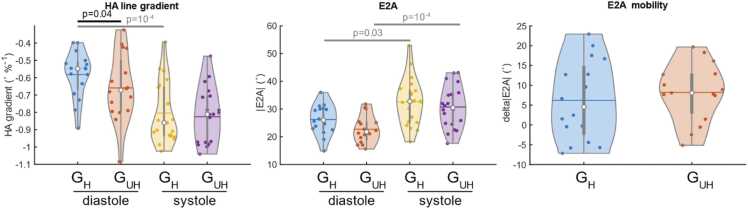
Violin plots of the cDTI angular measures compared between G_UH_ and G_H_ and between cardiac phases (apart from E2A mobility). E2A mobility is defined as the difference between systolic and diastolic |E2A|. *G_UH_* Ultrahigh strength gradients, *G_H_* High strength gradients, *cDTI c*ardiac diffusion tensor imaging, *E2A* absolute angle of second eigen vector

The cDTI angular measures are plotted in Fig. 7, showing a significant increase in the steepness of transmural HA line gradient (higher absolute value) at G_UH_ compared to G_H_ in diastole (−0.70 [0.17] vs. −0.55 [0.12] ˚/%, p = 0.04). |E2A| was not significantly different between G_UH_ and G_H_ nor was E2A mobility (systole – diastole).

**Fig. 8:**
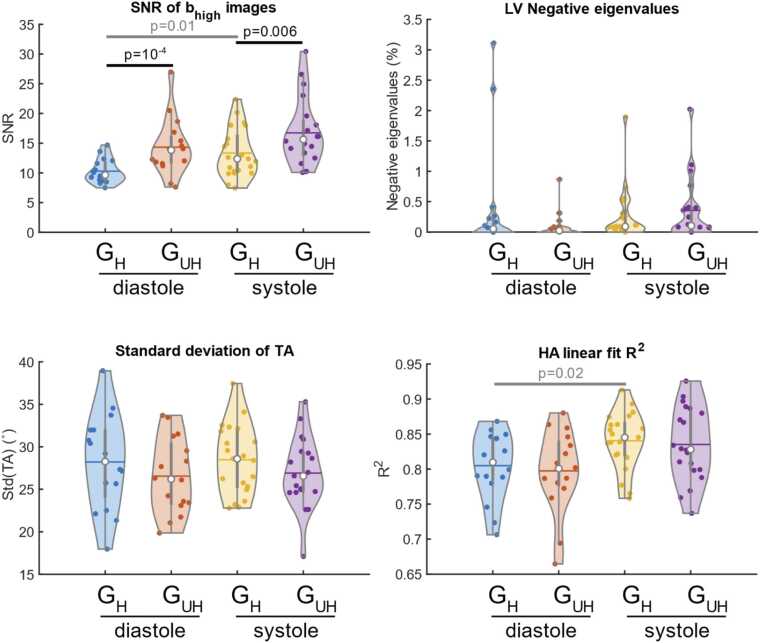
Violin plots comparing measures sensitive to cDTI data quality and SNR of the diffusion weighted images (b_high_=500 smm^−2^) between data acquired with G_UH_ and G_H_. Supplementary figure 4 shows the percentage of HA line profiles meeting the minimum criteria for inclusion in the HA line gradient comparison. *G_UH_* Ultrahigh strength gradients, *G_H_* High strength gradients, *cDTI c*ardiac diffusion tensor imaging, *SNR* signal to noise ratio, *HA* Helix angle

SNR of the b = 500 s/mm^2^ images was significantly higher at G_UH_ than G_H_ for both cardiac phases (12.9 [3.8] vs. 9.6 [2.9], p = 0.0002 diastole, 15.6 [5.9] vs. 12.5 [6.7], p = 0.006 systole) (Fig. 8). There were no significant differences between G_UH_ and G_H_ at either cardiac phase in the proportion of negative eigenvalues in the LV myocardium, R^2^ of the transmural variation in HA or the standard deviation of the transverse angle.

Given the relatively high rate of success for both systolic and diastolic cDTI, we performed a comparison of MCSE cDTI parameters between cardiac phases (see supplementary table 4).

MD was significantly higher in diastole than systole (1.64 [0.11] vs. 1.52 [0.24] × 10^−3^mm^2^/s, p = 0.002), when measured using G_UH_ but not with G_H_ (see Fig. 5). Tensor mode was significantly lower in diastole than in systole for both G_UH_ and G_H_ (0.16 [0.23] vs. 0.36 [0.17], p = 0.02 and 0.21 [0.12] vs. 0.35 [0.15], p = 10^−4^ respectively).

MD and tensor mode are calculated from the eigenvalues and the primary (E1) and secondary (E2) eigenvalue were significantly higher in diastole than systole when measured using G_UH_ (primary: 2.30 [0.13] vs. 2.11 [0.30] × 10^−3^mm^2^/s, p = 0.002; secondary: 1.58 [0.11] × 10^−3^mm^2^/s vs. 1.42 [0.23], p = 0.03). The secondary eigenvalue was also significantly higher in diastole in the data obtained with G_H_ (1.52 [0.13] vs. 1.40 [0.18] × 10^−3^mm^2^/s, p = 10^−4^) but the primary eigenvalue was not. The third eigenvalue was not significantly different between systole and diastole for either gradient strength.

For the angular measures, transmural HA line gradient was significantly steeper in systole than in diastole (−0.88 [0.23] vs. −0.55 [0.15] ˚/%, p = 10^−4^) when measured with G_H_, but not with G_UH_. In contrast, |E2A| was significantly higher in systole than in diastole when measured using both G_UH_ and G_H_ (G_UH_: 30.7 [7.3] vs. 21.3 [6.7]˚, p = 10^−4^; G_H_: 32.6 [10.9] vs. 26.0 [7.4]˚, p=0.03). The E2A mobility (systole – diastole) was 8.1 [9.9]˚ using G_UH_ and 4.6 [17.2]˚ using G_H_.

## 4 Discussion

Here, we provide the first comparison between cDTI performed using ultra-high gradient strength hardware and with more widely available high gradient performance in multiple cardiac phases. We have demonstrated an increase in the success rate of diastolic MCSE acquisitions when maximum gradient strength is increased from G_max_=66mT/m per axis to G_max_=116mT/m per axis from 73% to 80% and significant differences in transmural HA gradient between the G_H_ and G_UH_ data obtained in diastole, consistent with improvements in data quality at the higher maximum gradient strength. As a result of our high acquisition success rate, we were also able to provide a statistical comparison between cDTI parameters obtained at end systole and diastasis with MCSE in the largest cohort to date.

Our ultrahigh gradient strength system can potentially reach maximum gradient strengths of 300 mT/m per axis. However, our use of trapezoidal gradient waveforms with an equal gradient amplitude waveform design [11], [12] applied on two axes simultaneously resulted in a maximum gradient amplitude of 116 mT/m per axis due to stimulation limits. The use of a six-direction diffusion encoding scheme with gradients applied equally along two axes simultaneously, makes efficient use of the gradient strength, is common for in-vivo cDTI studies and provides a lower TE for more modest maximum gradient strengths. However, other encoding schemes should reach higher maximum gradient strength per axis. Higher maximum per axis gradient strengths could also have been achieved with higher b values, but we chose to use a set of b values close to those used for previous MCSE cDTI studies in order to avoid confounding the effects of b-value and maximum gradient strength on our results. The maximum per axis gradient strength of <120 mT/m also allows translation of our findings to the latest generation of commercially available ultrahigh strength gradient systems with maximum gradient strengths of around 200 mT/m per axis.

Afzali et al. [33] used the same scanner and compared second and third-order motion-compensated cDTI sequences with non-trapezoidal waveforms at b = 450 s/mm^2^ and b = 1000 s/mm^2^ during systolic contraction only. The use of non-trapezoidal waveforms and the use of a higher b-value meant that they were able to make more use of the ultrahigh maximum gradient strength of the Connectom scanner (G_max_∼295mT/m) at the expense of a longer TE of 74 ms (for second-order motion compensation at b = 1000 s/mm^2^). Afzali et al.’s [33] results demonstrated small differences in MD between data acquired with second and third-order motion compensation at both b values. While increases in motion compensation order result in inevitable increases in TE, an exploration of the ability of third and higher order motion compensation in minimizing motion-related artefacts in diastolic MCSE is a logical extension of our work for the future.

Our success rates for diastolic MCSE were higher than we have found in previous studies [24], [25], [26], albeit with lower maximum gradient strengths previously. In Scott et al. [24], we used a 3T scanner with maximum gradient strength of 45 mT/m per axis, at a maximum b-value of 450 s/mm^2^, providing a TE = 76ms for second-order MCSE (similar field of view and EPI readout) which resulted in a success rate of 53% (8/15) in healthy volunteers. The same protocol on the same scanner in patients with hypertrophic cardiomyopathy resulted in a success rate of 40% (4/10) subjects in diastole [25]. More recently, on a 3T scanner with 60 mT/m maximum gradient strength per axis (achieved 54 mT/m), resulting in TE = 62 ms, diastolic MCSE was successful in 40% of healthy volunteers (8/20) [26]. Even when we limited G_max_ to 80 mT/m and achieved G_max_=66 mT/m per axis (G_H_), we achieved a 73% success rate in this work, suggesting that, while improvements in robustness of diastolic MCSE cDTI are possible at ultrahigh gradient strengths, perhaps the biggest benefit for the diffusion weighting and second-order MCSE design used here is observed between 60 mT/m and 80 mT/m per axis. For a given b value, readout and slew rate, the minimum TE reduces rapidly while the flat top duration of the diffusion encoding forms a substantial proportion of the TE, but approaches a plateau as the diffusion encoding gradient approach triangular shapes. Further limitations to the TE reduction with increasing G_max_ occur due to rise time limitations imposed by gradient hardware and stimulation limits. As described above, larger proportional reductions in TE would be expected for higher b-values [33], [39] and future work might consider the optimal b-values for MCSE cDTI acquisitions as has been investigated for DTI in general and in application to the heart [40], [41].

The reduced robustness of second-order MCSE cDTI in diastole compared to systole is in accordance with our previous findings [24], [25], [26]. The cardiac phase of choice for many CMR techniques is diastasis (e.g., late gadolinium enhancement imaging, coronary angiography, parametric mapping, anatomical imaging). However, in these techniques minimizing the magnitude of motion during image encoding is the key step in avoiding image blurring and ghosting. cDTI requires that the motion during diffusion encoding does not result in substantial bulk motion related signal loss, while the image encoding is typically assumed to be robust to motion with the rapid single shot acquisition schemes and modest spatial resolutions currently in use. Second-order motion-compensated spin-echo cDTI relies on the assumption that motion during the diffusion encoding is well modeled by motion terms up to the second order (constant acceleration within each voxel). Any higher-order motion that occurs during encoding (i.e., third order and above) will result in phase dispersion across a voxel and, therefore, artefactual signal loss. As a result, myocardial motion with constant acceleration can have large amplitudes and result in good quality MCSE cDTI data quality (e.g., during systolic contraction), while low amplitude high order motion could result in severe motion-related artefacts in MCSE cDTI (e.g., diastasis). The opposite would be true for CMR techniques where motion during the image encoding causes artefacts.

The increase in SNR that we measured in both cardiac phases when increasing the maximum gradient strength was to be expected due to the reduction in echo time at higher maximum gradient strength. This SNR increase resulted in a significant increase in the subjective image quality score in diastole from G_H_ to G_UH_ (p = 0.04), with a substantial increase in the proportion of datasets scored the highest value of 3 (3 vs. 7). However, the differences in other parameters were less predictable. In our results, increasing from G_H_ to G_UH_ results in a significant reduction in FA in systole and an increase in the steepness of the transmural HA gradient in diastole. The increase in SNR with higher available maximum gradient strength is due to the reduction in TE. Assuming a myocardial T2 = 47 ms [42], the reduction in TE = 58 ms at G_H_ to TE = 48 ms at G_UH_ would result in an increase in SNR by 24%. We measured increases of 34% in diastole and 25% in systole. There are known systematic errors in cDTI parameters due to the effects of noise [40], [41], [43]. With a reduction in SNR, the increased noise on average results in an increase in the difference between eigenvalues, and, hence an increase in FA, sometime referred to as eigenvalue repulsion. The primary and secondary eigenvalue were marginally higher for G_UH_ than G_H_ in systole (primary: 2.14 [0.25] vs. 2.11 [0.24] × 10^−3^ mm^2^/s, p = ns; secondary: 1.42 [0.17] vs. 1.39 [0.17] × 10^−3^ mm^2^/s, p = ns) while the third eigenvalue was significantly increased using G_UH_ (1.04 [0.16] vs. 1.006 [0.098] × 10^−3^ mm^2^/s, p = 0.03) resulting in the observed significant decrease in FA at G_UH_ over G_H_.

We also measured a significant increase in the magnitude of the transmural HA gradient in diastole at G_UH_ compared with G_H_. These results are consistent with a reduction in data quality at lower G_max_ as a loss of diffusion encoding, e.g., caused by a reduction in SNR, would generally push the HA results towards a zero gradient. We also observe a significantly steeper (more negative) HA line gradient in systole than diastole using G_H_, but this difference is not observed with G_UH_. While using STEAM acquisitions, we have measured steeper HA line gradients in systole than in diastole [24], [26], differences measured with MCSE have been less [26], [31] and differences between the two states measured histologically are negligible [2], suggesting that the true difference is probably small. As a result, in the absence of a difference between cardiac phases in HA line gradient at the higher G_max_ value, we attribute the difference at G_H_ to a loss of diastolic data quality at G_H_ compared to G_UH_.

We provide the most comprehensive comparison between cDTI parameters obtained in multiple cardiac phases using spin echo-based sequences. Moulin et al. [31] used a second-order MCSE sequence acquired during free breathing in early systole, late systole, and end diastole on a scanner with maximum gradient strength of 80 mT/m per axis, with success rates of 9/9 in early systole, 7/9 in late systole, and 6/9 at end diastole. In our 14 successful complete datasets, we demonstrated significant reductions in the primary and secondary eigenvalues between diastole and systole, resulting in a significant reduction in MD, which was only detectable using G_UH_. This diastolic elevation in MD is consistent with results observed using STEAM [44], with the patterns demonstrated in our previous studies in MCSE with lower success rates [24] and with those of Moulin et al. [31]. A reduction in the number of restrictions or hinderances to diffusion would result in a higher measured diffusivity as would an increase in SNR in the low SNR regime. As we observe a lower SNR in diastole, it seems unlikely that the reduced MD in this cardiac phase is a consequence of noise effects, but rather a reflection of changes in the underlying microstructure during cardiac contraction. The use of G_UH_ also enabled the detection of a significant increase in tensor mode between diastole and systole, reflecting a change from a more stick-like tensor towards a more disk-like tensor in systole than diastole, consistent with our previous findings [24].

In addition to differences in image quality between results obtained with the two G_max_ values, the use of G_UH_ also shortens the diffusion encoding duration which results in a shorter effective diffusion time. While the unequal bipolar gradients used in the second-order motion-compensated encoding employed here do not have a unique diffusion time, in supplementary figure 5, we demonstrate that there are two effective diffusion times for this gradient design. At G_H_ these diffusion times were 13.5 ms and 20.0 ms and at G_UH_ they were 10.3 ms and 15.0 ms. At shorter diffusion encoding times, water molecules travel shorter distances, interacting with fewer microstructural hinderances and restrictions resulting in lower measured FA and higher measured MD.

In this work, we show that MCSE acquisitions using high and ultrahigh gradient strength can measure significant differences in |E2A| between cardiac phases, which has been validated as a measure of sheetlet orientation when measured with STEAM [2]. While the magnitude of E2A mobility is lower in magnitude than observed for STEAM in healthy volunteers (c.f. 45 [11]˚ [2]) this measured change using MCSE is consistent with the results of Moulin et al. [31]. This finding requires further investigation to determine whether MCSE can match the sensitivity of STEAM to changes in sheetlet function in pathology and where the two orders of magnitude difference in diffusion time between the two sequences may result in complimentary microstructural information.

Despite the increased success of diastolic MCSE acquisitions at ultrahigh gradient strength and the robustness of systolic MCSE acquisitions at both high and ultrahigh maximum gradient strength, further technical developments are required. The robustness of diastolic MCSE acquisitions remains a barrier to clinical translation of the method, which would likely require success rates upwards of 95%. Strategies for further increases in the success rate may include selecting diastolic trigger delays for MCSE cDTI based on long-axis cine data in addition to the short-axis images used here to account for through plane motion and the use of MCSE trigger scout acquisitions [31]. We did, however, attempt to adjust the trigger delay for diastolic acquisitions where the initial breath holds demonstrated motion-induced signal loss. Future work may trade the increase in SNR enabled by ultrahigh strength gradient systems for increased spatial resolution, which may deliver improved resilience to motion due to reduced intravoxel dephasing. Alternatively, methods such as principal component analysis temporal maximum intensity projection (PCA-TMIP) [45] have been used to combine cDTI data from multiple trigger delays where signal loss may be localized and trigger delay dependent.

## 5 Limitations

Our cohort was relatively young and comprised solely of a control population, albeit with a range of habitus from underweight (BMI 17.5 kg/m^2^) to obese (BMI 30.6 kg/m^2^) and a range of heart rates (46–79 BPM). Further assessment in patients is required for a true determination of robustness and to assess the sensitivity of MCSE derived |E2A| to pathological changes in microstructural dynamics. While we have found that with careful explanation of the process, patients with a wide range of conditions tolerate cDTI acquisitions as part of long research protocols, our previous work in healthy volunteers [24] and hypertrophic cardiomyopathy patients [25] demonstrated a slightly lower success rate (40% vs. 53%) for diastolic MCSE acquisitions at G_max_= 43 mT/m.

Our cohort was of a sufficient size to detect significant differences in transmural HA gradient and FA between G_UH_ and G_H_. However, the differences we measured between cDTI parameters between the systole and diastole detected only using G_UH_ (e.g., MD and primary eigenvalue) suggest that further significant differences between G_H_ and G_UH_ may be detected with larger cohorts.

We used a relatively conservative protocol with a lower spatial resolution than some other MCSE protocols [18], [31], minimal encoding directions, long acquisition durations, single-slice imaging, and breath holding. However, this was based on a protocol that we have used with STEAM to demonstrate pathological changes in the microstructural dynamics in a range of heart diseases and its use here means that our results are more amenable to comparisons with prior findings [2], [3], [13], [14].

## 6 Conclusion

MCSE performed on ultrahigh gradient strength systems provides high success rates for diastolic and systolic cDTI and increases in SNR over high gradient strength acquisitions. While the robustness of diastolic MCSE will need to improve further for routine clinical use, ultrahigh gradient strength systems do result in higher success rates than high gradient strength systems and cDTI results are more sensitive to changes in microstructure, such as those between cardiac phases shown here. The increasing availability of ultrahigh-strength gradient hardware in commercially available clinical whole-body systems will contribute towards greater use of cDTI in clinical research and eventual translation into the clinic.

## Author contributions

**Shubhajit Paul:** Writing – review & editing, Software, Methodology, Investigation, Formal analysis, Data curation, Conceptualization. **Camila Munoz:** Writing – review & editing, Methodology, Investigation. **Pedro F. Ferreira:** Writing – review & editing, Visualization, Validation, Software, Funding acquisition, Conceptualization. **C.John Evans:** Writing – review & editing, Resources, Project administration, Methodology, Investigation, Conceptualization. **Sonya Foley:** Writing – review & editing, Supervision, Project administration. **Fabrizio Fasano:** Writing – review & editing, Resources, Methodology. **Derek K. Jones:** Writing – review & editing, Supervision, Resources, Project administration, Methodology. **Dudley J. Pennell:** Writing – review & editing, Supervision, Resources, Project administration, Methodology, Conceptualization. **Sonia Nielles-Vallespin:** Writing – review & editing, Supervision, Methodology, Funding acquisition, Conceptualization. **Andrew D. Scott:** Writing – review & editing, Writing – original draft, Validation, Supervision, Software, Project administration, Methodology, Investigation, Funding acquisition, Formal analysis, Data curation, Conceptualization.

## Declaration of competing interests

The authors declare the following financial interests/personal relationships which may be considered as potential competing interests: Andrew Scott reports that financial support was provided by Siemens Healthineers AG. Dudley Pennell reports that financial support was provided by Siemens Healthineers AG. Sonia Nielles-Vallespin reports that financial support was provided by Siemens Healthineers AG. Fabrizio Fasano reports that financial support was provided by Siemens Healthineers AG. The remaining authors declare that they have no known competing financial interests or personal relationships that could have appeared to influence the work reported in this paper.

## Data Availability

Data will be provided on reasonable application to the corresponding author subject to ethical approval constraints and institutional agreements.
