# Supplementary material for: Motion-compensated spin-echo cardiac diffusion tensor imaging in multiple cardiac phases using an ultrahigh gradient strength scanner
Source: J Cardiovasc Magn Reson. 2026 Jan 29;28(1):102699. doi: 10.1016/j.jocmr.2026.102699 (PMC13207544; doi:10.1016/j.jocmr.2026.102699)
Supplement: Supplementary file 1 — Supplementary material [file mmc1.pdf]

Supplementary table 1

Gradient scheme label	Maximum gradient strength per axis allowed (mT/m)	Maximum gradient strength per axis specified in design compatible with non-zero flat top time in M2 compensated gradients (mT/m)	Maximum gradient strength per axis achieved (mT/m)	Maximum gradient strength achieved from two simultaneously applied gradients (mT/m)	Slew rate achieved per axis (T/m/s)
G_H	80	80	66	93	83
G_{UH}	300	116	116	164	64

Supplementary table 2: "Total encoding time" is the time from the beginning of the first diffusion encoding gradient lobe to the end of the last, incorporating 8 ramp times, 2 short flat tops, 2 long flat tops and the gap.

Gradient scheme label	Maximum gradient strength per axis run (mT/m)	Gradient ramp up/down time (ms)	Flat top time 1 (ms)	Flat top time 2 (ms)	Gap Duration (ms)	Total encoding time (ms)	180˚ pulse duration (ms)
G_H	**66**	**0.8**	**5.3**	**11.4**	**6.9**	**46.7**	**2.2**
G_{UH}	**116**	**1.8**	**3.0**	**6.8**	**5.6**	**39.6**	**2.2**

Supplementary table 3

A summary of DTCMR parameters obtained at peak systole and diastasis in the acquisitions deemed successful in the study cohort. Parameters were measured as the mean over the LV myocardium apart from |E2A| which was calculated as the median. Where a Shapiro-Wilks test suggested a non-normal distribution for either maximum gradient strength values are shown as median [IQR] otherwise as mean±SD. Paired statistical comparisons were made between the data acquired with G_{UH} (116mT/m) and G_H (80mT/m) using a t-test for data considered normal and a Wilcoxon sign-rank test otherwise. Comparisons where $p<0.05$ are indicated with an asterisk *.

Data from acquisitions performed with similar protocols in prior studies with maximum gradient strengths of 43mT/m[1] and 60mT/m[2] (labelled as 54mT/m to be consistent with the labelling of the G_H and G_{UH} data here, as that was the maximum gradient strength achieved) is provided for reference. Acquisition success rate is provided as n=successful/total acquisitions. Mode was not quoted in reference [2].

Parameter	**Diastole**				**Systole**			
	43mT/m n=8/15	*54mT/m n=8/20*	G_H: 64mT/m n=16/22	G_{UH}: 116mT/m n=16/20	*43mT/m n=14/15*	*54mT/m n=19/20*	G_H: 64mT/m n=22/22	G_{UH}: 116mT/m n=21/21
MD (x10^{-3} mm^{2}s^{-1})	*1.78 [0.34]*	*1.66±0.16*	1.56 [0.08]	1.66 [0.11]	*1.46 [0.43]*	*1.51±0.14*	1.50 [0.17]	1.52 [0.19]
FA	*0.41 [0.07]*	*0.346 [0.062]*	0.387 [0.026]	0.356 [0.044]	*0.40 [0.09]*	*0.330 [0.050]*	0.373 [0.019]*	0.349 [0.040]*
Mode	*0.29 [0.07]*	-	0.22 [0.13]	0.18 [0.22]	*0.36 [0.17]*	-	0.34 [0.14]	0.35 [0.19]
HALG (° %$^{-1}$)	*-0.45 [0.19]*	*-0.66±0.21*	-0.55 [0.12]*	-0.70 [0.17]*	*-0.77 [0.09]*	*-0.81 [0.16]*	-0.88 [0.23]	-0.81 [0.26]
|E2A| (°)	*26 [5]*	*21.0 [7.8]*	26.0 [7.8]	21.2 [7.3]	*37 [9]*	*35.4 [5.5]*	33 [12]	30 [11]
E1 (x10^{-3} mm^{2}s^{-1})	*2.59 [0.30]*	*2.26±0.15*	2.21 [0.13]	2.29 [0.12]	*2.08 [0.43]*	*2.06 [0.14]*	2.11 [0.24]	2.14 [0.25]
E2 (x10^{-3} mm^{2}s^{-1})	*1.67 [0.31]*	*1.59±0.17*	1.49 [0.14]	1.58 [0.11]	*1.34 [0.43]*	*1.43 [0.15]*	1.39 [0.17]	1.42 [0.17]
E3 (x10^{-3} mm^{2}s^{-1})	*1.07 [0.41]*	*1.07 [0.29]*	0.981 [0.071]	1.09 [0.17]	*0.94 [0.41]*	*1.05 [0.19]*	1.006 [0.098]*	1.04 [0.16]*

Supplementary table 4

Comparison of DTCMR parameters between cardiac phases. Values are quoted as in supplementary table 1, with comparisons in this case between acquisitions at peak systole and end diastole at the same G_{max}.

Parameter	G_H		G_{UH}	
	Diastole	Systole	Diastole	Systole
MD ($x10^{-3}$ mm^2s^{-1})	1.56 [0.13]	1.51 [0.19]	1.67[0.11]*	1.52[0.24]*
FA	0.381[0.026]	0.374 [0.018]	0.359 [0.048]	0.355 [0.030]
Mode	0.21 [0.12]*	0.35 [0.15]*	0.16 [0.23]*	0.36 [0.17]*
HALG (˚ $\%^{-1}$)	-0.55 [0.15]*	-0.88 [0.23]*	-0.68 [0.21]	-0.81 [0.24]
|E2A| (˚)	26.0 [7.4]*	32.6 [10.9]*	21.3 [6.7]*	30.7 [7.3]*
E1 ($x10^{-3}$ mm^2s^{-1})	2.21 [0.14]	2.14 [0.24]	2.30 [0.13]*	2.11 [0.30]*
E2 ($x10^{-3}$ mm^2s^{-1})	1.49 [0.16]*	1.39 [0.18]*	1.58 [0.11]*	1.42 [0.23]*
E3 ($x10^{-3}$ mm^2s^{-1})	0.98 [0.10]	0.9999[0.098]	1.10 [0.10]	1.03 [0.18]
ΔE2A (˚)	4.6 [17.2]		8.1 [9.9]	

Supplementary figure 1: Example SNR maps averaged over the encoding directions for b=500smm^{-2} data. The subject corresponds to the subject shown in figure 2 in the main manuscript. The clear increase in SNR at the higher G_{max} value is evident.

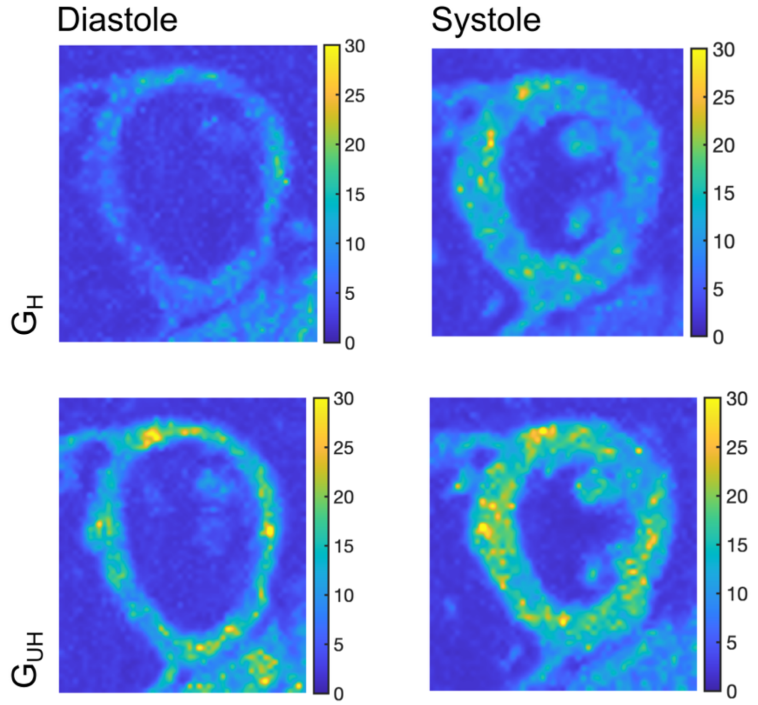

Supplementary figure 2: Examples of diastolic diffusion weighted images from an acquisition where the HA map scored 3 (upper) and scored 0 (lower). In both cases 6 repetitions are shown and 2 (the same) encoding directions. The dataset where HA maps scored 0 has two frames that were manually excluded from the tensor calculation due to complete myocardial signal loss. Several other images show regional signal loss artefacts (red arrows) in the septal wall, but the images were not excluded from the tensor calculation in the hope that the diffusion tensor in the rest of the heart could be adequately calculated. Both datasets were acquired using G_H.

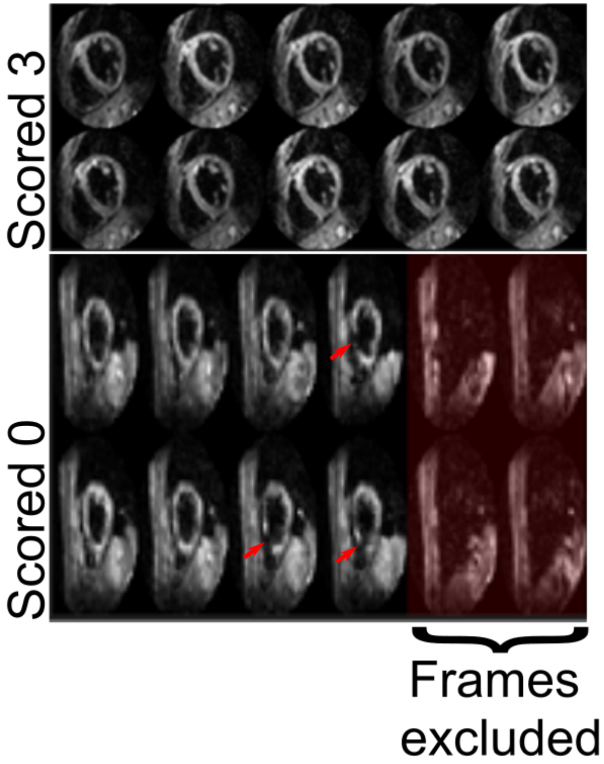

Supplementary figure 3: Examples of HA maps scored 3 to 0 acquired using G_{UH} in diastole.

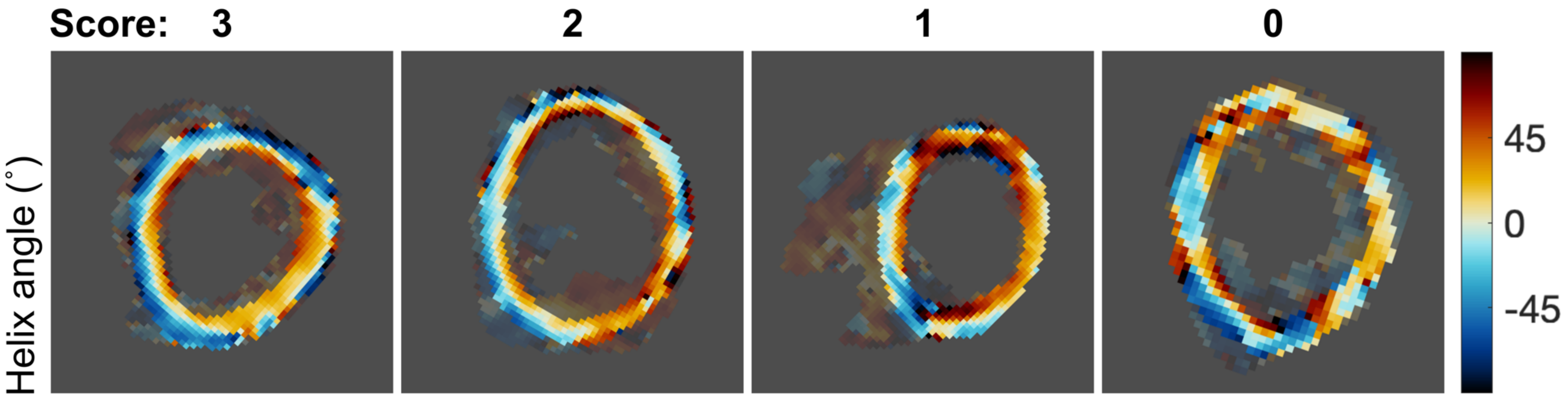

Supplementary figure 4: The proportion of HA line profiles meeting the minimum quality criteria for inclusion in the HA line gradient comparison shown in figure 7. The minimum criteria are a negative slope and an $R^2>0.4$. There were no significant differences between G_H and G_{UH} datasets.

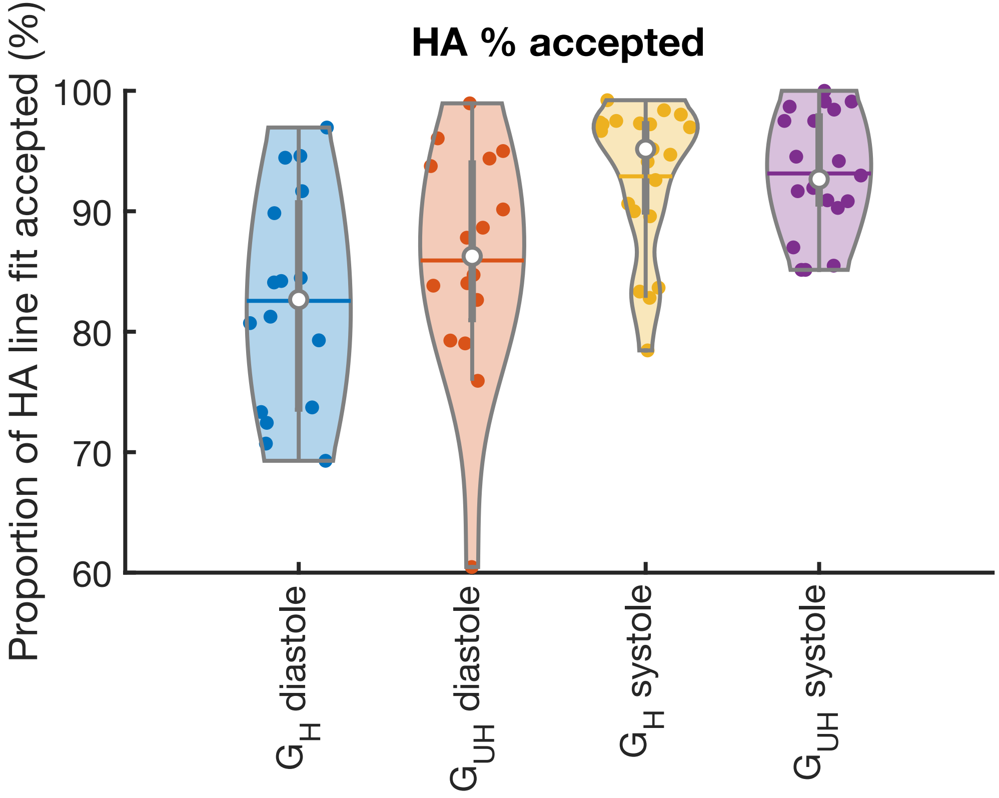

Supplementary figure 5: The single axis zeroth gradient moment and cumulative b value for the G_{UH} and G_H gradient waveforms used. The effective diffusion times for GH are approximately 13.5ms and 20.0ms with the longer diffusion time contributing approximately 55% of the total b value. For the GUH waveforms, the effective diffusion times are 10.3ms and 15.0ms with the longer diffusion time contributing approximately 55% of the b value.

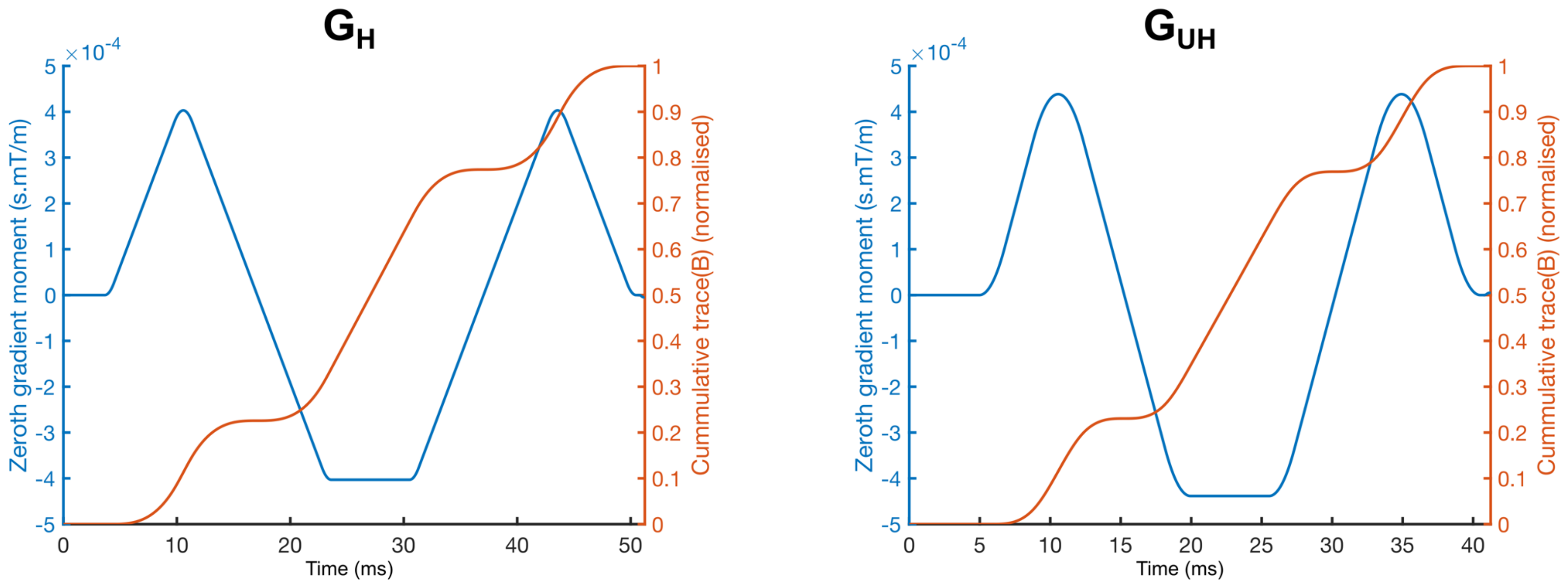

References

1. Scott AD, Nielles-Vallespin S, Ferreira PF, et al. An in-vivo comparison of stimulated-echo and motion compensated spin-echo sequences for 3 T diffusion tensor cardiovascular magnetic resonance at multiple cardiac phases. *J Cardiovasc Magn Reson*. 2018;20(1). doi:10.1186/s12968-017-0425-8

2. Scott AD, Wen K, Luo Y, et al. The effects of field strength on stimulated echo and motion compensated spin echo diffusion tensor cardiovascular magnetic resonance sequences. *J Cardiovas Magn Reson*. June 2024:101052. doi:10.1016/J.JOCMR.2024.101052
